# Pharmacokinetics, Pharmacodynamics, Safety and Immunogenicity of CMAB807, a New Denosumab Biosimilar, in Healthy Chinese Subjects

**DOI:** 10.3389/fphar.2022.821944

**Published:** 2022-01-24

**Authors:** Hanjing Chen, Weili Chen, Fei Yuan, Qingcheng Guo, Xunmin Zhang, Chenguang Wang, Xuening Li

**Affiliations:** ^1^ Department of Clinical Pharmacology, Zhongshan Hospital, Fudan University, Shanghai, China; ^2^ Taizhou Mabtech Pharmaceuticals Co., Ltd, Taizhou, China

**Keywords:** denosumab, biosimilar, pharmacokinetics, pharmacodynamics, immunogenicity, osteoporosis, RANKL

## Abstract

**Objective:** Pharmacokinetics (PK), pharmacodynamics (PD), safety and immunogenicity studies were conducted to evaluate the bioequivalence of CMAB807, a biosimilar to denosumab (Prolia^®^), which is the only approved RANKL inhibitor for the treatment of osteoporosis.

**Methods:** In this randomized, double-blind, single-dose phase I study, 132 healthy Chinese male subjects received a subcutaneous injection of 60 mg of CMAB807 or denosumab at a 1:1 ratio. The PK, PD, safety and immunogenicity results were assessed prior to and up to 126 days after administration.

**Results:** The PK profiles of CMAB807 and denosumab were similar. The geometric mean ratios of the maximum concentration (C_max_), AUC_0-t_ and AUC_o-∞_ were 102.41, 104.15 and 103.89%, respectively, and the 90% confidence interval was observed to be within 80.00–125.00%, which indicated the bioequivalence of CMAB807 and denosumab. The PD profiles of the two groups were also comparable. The production of the C-terminal cross-linking telopeptide of type I collagen (CTX1) was inhibited by up to 85% for 10 days, and this inhibition was sustained for up to 126 days in both the CMAB807 and denosumab groups. No subjects in the CMAB807 group, three subjects in the denosumab group before administration, and two subjects in the denosumab group after administration were positive for anti-drug antibody (ADA). Adverse events (AEs) were observed in 98.5% of subjects in both groups. The most common AE recorded was increased parathyroid hormone (PTH) levels, with incidences of 92.4 and 95.5% in the CMAB807 and denosumab groups, respectively. No clinically meaningful differences were observed in safety and immunogenicity between CMAB807 and denosumab.

**Conclusion:** CMAB807 represents a new potential treatment option for patients with osteoporosis.

C**linical Trial Registration:** https://clinicaltrials.gov (Registration No. NCT03925051), http://www.chinadrugtrial/org.cn/index.html (Registration No. CTR20190800).

## Introduction

Denosumab is a fully human IgG κ-type monoclonal antibody (mAB) that binds to receptor activator of nuclear factor-κB ligand (RANKL) (Tanaka et al., 2005). This binding prevents the activation of RANK and inhibits the formation, activation and survival of osteoclasts, thus reducing the number and activity of osteoclasts and subsequently resulting in a reduction in bone resorption and an increase in the bone mass and strength of cortical bone and trabecular bone (Deeks, 2018). Denosumab (Prolia^®^) was approved by the Food and Drug Administration (FDA) as a treatment to increase bone mass in postmenopausal women with osteoporosis and men with osteoporosis who are at high risk of fractures (Amgen, 2021). However, the high price of denosumab makes it difficult for patients in developing countries such as China to afford.

Biosimilars can improve the health of patients by increasing their accessibility to biological molecules due to decreasing healthcare-associated costs (Mulcahy et al., 2018). Denosumab biosimilars are being actively developed worldwide (Zhang et al., 2020; Khan, 2021; Singh et al., 2021; Zhang et al., 2021). However, no denosumab biosimilar has been marketed in China to date. CMAB807, a recombinant anti-RANKL human mAB injection, is a candidate biosimilar of denosumab developed according to Chinese biosimilar guidelines (NMPA, 2015) and current FDA and European Medicines Agency (EMA) guidelines for the development of biosimilar products (EMA, 2015; FDA, 2015). The similarity of CMAB807 to the reference product was established through a stepwise approach of physicochemical and biological characterization and preclinical comparability experiments (unpublished data).

Comparative pharmacokinetics (PK) studies designed to document similar PK profiles for key parameters of biosimilar and reference medicinal products are an essential component of biosimilar development programs. The primary endpoints are usually PK parameters, such as the area under the curve (AUC) and maximum concentration (C_max_) (NMPA, 2015). Serum concentration-time profiles of denosumab were best characterized by a two-compartment model with first-order absorption and parallel linear and nonlinear elimination (FDA, 2010). In the present study, the PK profile of 60 mg of denosumab was compared with that of CMAB807 to evaluate the bioequivalence of the two products. The pharmacodynamics (PD) profiles, safety and immunogenicity were also evaluated.

## Methods

### Study Design and Study Participants

We conducted a phase I, double-blind, randomized, parallel-group, single-dose study. One hundred thirty-two 132) healthy Chinese male subjects were planned to be enrolled. This study was performed at the Department of Clinical Pharmacology, Zhongshan Hospital, Fudan University, Shanghai, China, from June 2019 to March 2020. The protocol, informed consent and amendments were approved by the Medical Ethics Committee of Zhongshan Hospital, Fudan University. All subjects signed written informed consent forms before participating. The study was registered at ClinicalTrials.gov (Registration No. NCT03925051) and the Chinese Clinical Trial Registry (Registration No. CTR20190800). The study was conducted according to the guidelines for Good Clinical Practice and the ethical standards for human experimentation established in the Declaration of Helsinki.

Eligible subjects were healthy Chinese male subjects aged 18–65 years with a body weight greater than 50 kg (body mass index (BMI) of 19–26 kg/m^2^). They were deemed healthy after assessments of physical examinations, vital signs, medical history, laboratory safety tests, and 12-lead electrocardiogram (ECG) measurements (QTc<450 ms). In addition, their blood calcium levels were between 2.15 and 2.55 nmol/L (including the borders).

Exclusion criteria included a history of clinically significant allergies, osteomyelitis or osteonecrosis of the jaw, odontopathy or maxillary disease in the active stage or in remission, hyperthyroidism or hypothyroidism, rheumatoid arthritis, osteomalacia, Paget’s disease, lumbar disc herniation, or mental disease. Subjects were excluded if they were positive for hepatitis B surface antigen, human immunodeficiency virus, or hepatitis C antibodies; consumed more than eight cups of caffeine-containing drink per day; smoked more than five cigarettes per day; consumed over 14 servings of alcohol per day; were vaccinated with a live attenuated virus vaccine; lost more than 400 ml of blood; or who had taken any drugs, vitamins or herbal formulations within 30 days.

Subjects were screened 2–30 days prior to the administration of the study drug. Eligible subjects were admitted to the hospital the day before administration. A random table was generated by unblinded statisticians using random blocks with SAS 9.4 statistical analysis software (Cary, NC, United States). Eligible subjects were sorted according to their weights and assigned to the denosumab or CMAB807 group in a 1:1 ratio to minimize interindividual differences in each group. A single dose of CMAB807 (60 mg/1 ml, batch number: 201903001, Shanghai Biomabs Pharmaceutical Co., Ltd., protein concentration: 62.24 mg/ml) was administered to the CMAB807 group, and denosumab (60 mg/1 ml, batch number: 1093571, Amgen, Thousand Oaks, California, United States, protein concentration: 60.6 mg/ml) was administered to the denosumab group subcutaneously within 10 cm of the periumbilical region. Subjects remained in the hospital for 3 days after administration and were followed up on days 4–126.

### PK Evaluations

Blood samples were collected for PK analysis within 30 min prior to dosing (predose) and at 6 h, 2, 3, 4, 6, 8, 10, 12, 14, 18, 21, 28, 35, 42, 56, 70, 84, 112, and 126 days after dosing. Tubes containing whole blood (3 ml) were placed at room temperature for at least 30 min to induce coagulation and centrifuged promptly at 1921 × g at 4°C for 10 min. Shortly after the separation of the whole blood, serum was carefully transferred to another tube and stored at −70°C until shipment to United-Power Pharma Tech Co., Ltd. for analysis using an electrochemiluminescence assay (ECLA). The overall assay accuracy (% deviation from nominal) was -13.9–13.1%, and the precision (% coefficient of variation) was 7.1–16.2%. Both CMAB807 and Prolia^®^ were confirmed to be stable at -90∼-60 °C for 6 months, at -30∼-10 °C for 3 months, at room temperature for 1 h and 10 min, and after five freeze-thaw cycles. Linear calibration curves were obtained in the range of 25.0–6,400 ng/ml, with a lower limit of quantitation (LLOQ) of 25.0 ng/ml and an upper limit of quantitation (ULOQ) of 6,400 ng/ml. Values below the LLOQ were treated as 0 when observed before C_max_ and were excluded from the PK analysis when observed after C_max_.

The primary PK endpoints were C_max_, AUC_0-t_ and AUC_0-∞_. Other parameters assessed for denosumab included the time to reach the maximum concentration (T_max_), apparent terminal t_1/2_, apparent total plasma clearance (CL/F) and volume of distribution (Vz/F), which were measured as secondary PK parameters. All PK parameters were calculated with a noncompartmental analysis (NCA) using Phoenix^®^ WinNonlin^®^ software (version 8.0, Certara L.P., Princeton, NJ, United States). The actual sample collection times were considered for the PK analysis. C_max_ and T_max_ were obtained visually from the concentration-time curve. The AUC_0-∞_ was determined by adding AUC_0–t_ to the extrapolated area, which was calculated using the linear trapezoidal method.

### PD Evaluations

Serum samples used to measure denosumab pharmacodynamics were obtained predose, 6 h, and 3, 10, 28, 56, 84, 112, and 126 days postdose in the fasted state in the morning. Two milliliters of whole blood were collected, centrifuged and stored until PK sample procedures were performed. The serum C-terminal cross-linking telopeptide of type I collagen (CTX1) concentrations were analyzed by United-Power Pharma Tech Co., Ltd. using an electrochemiluminescence immunoassay (ECLIA). Concentrations ranging from 0.010 to 6.00 ng/ml can be accurately detected using the validated method. Serum CTX1 concentrations below the LLOQ (0.010 ng/ml) were set to 0.010 ng/ml before data analysis. The actual sample collection times were also considered for the PD analysis. The concentration-time data included the minimum observable serum CTX1 concentration (I_min_), the observed time of the lowest serum CTX1 concentration (T_min_), the observed highest inhibition percentage of CTX1 (I_max_), the AUC from zero to 126 days (AUEC_0-126d_), and the final quantifiable CTX1 concentration (AUEC_0-t_). Additionally, the percent change in the CTX1 concentration from baseline between the two groups was compared visually.

### Immunogenicity Evaluations

Blood samples (4 ml) were collected for the immunogenicity evaluation at predose and 28, 56, 84, 112, and 126 days after dosing. The anti-drug antibody (ADA) samples were analyzed at United-Power Pharma Tech Co., Ltd. using the bridging electrochemiluminescence immunoassay (Bridging-ECLIA) and the MSD Discovery Workbench (v 4.0.12).

### Safety Evaluations

Clinical safety evaluations were conducted by performing physical examinations and assessing vital signs, 12-lead ECGs, and immunogenicity. Laboratory safety evaluations were conducted and included routine hematology (including hematocrit, hemoglobin, platelet count and white blood cell count), serum chemistry (including alanine aminotransferase, aspartate aminotransferase, alkaline phosphatase, γ-glutamyltranspeptidase, total protein, albumin, total bilirubin, direct bilirubin, creatinine, urea, uric acid, glucose, triglyceride, total cholesterol, high-density lipoprotein cholesterol (HDL-C), low-density lipoprotein cholesterol (LDL-C), potassium, sodium, calcium, magnesium, phosphorus and chloride), coagulation functions (including the prothrombin time (PT), activated partial thromboplastin time (APTT), international normalized ratio (INR), and thrombin time (TT)), thyroid functions (including thyroid stimulating hormone (TSH), free triiodothyronine, free thyroxine, triiodothyronine, thyroxine and parathyroid hormone (PTH)) and urinalysis (including protein, glucose, ketones, white blood cells, blood, pH and gravity). Both clinical and laboratory safety evaluations were performed before and at certain time points after administration. Adverse events (AEs) were recorded and graded according to the Common Terminology Criteria for Adverse Reactions, V. 5.0 (CTCAE 5.0) of the National Cancer Institute and were evaluated for intensity, duration, outcome, and relationship to the study drug by the investigators. Investigators monitored AEs throughout the study.

### Estimation of Sample Size

Sample size calculations were performed using PASS (version 16.0.2, NCSS, Kaysville, UT, United States), assuming that C_max_ and AUC were the primary endpoints and AUC_0-∞_, T_max_, and t_1/2_ were the secondary endpoints. The coefficient of variation (CV) of the PK parameters was predicted to be 32% based on data obtained from healthy volunteers (Bekker et al., 2004). One hundred sixteen subjects (58 per group) needed to complete the study to achieve 90% power (1-β) at the 5% nominal level (α = 0.05, two one-sided tests) such that the 90% confidence intervals (CIs) for the geometric mean ratio (GMR) fell within the acceptance criterion of 80.00–125.00%. Allowing for 10% potential drop-outs, the sample size was defined to be 132 (66 per group).

### Statistical Analysis

All subjects who were treated with the study drug were included in the safety analysis set. Subjects who were administered the study drug and had at least one PK or PD parameter collected were included in the PK or PD set populations, respectively. In addition, subjects whose drug concentration before administration was greater than 5% C_max_ were eliminated from the PK analysis set.

All statistical tests were conducted using SAS 9.4 (SAS Institute Inc., Cary, NC, United States). A descriptive statistical analysis was conducted for the PK and PD parameters and the demographic data. The comparisons were performed using the chi-square test for categorical variables, Student’s t-test for normally distributed data, or the Wilcoxon signed-rank test for data with an unknown distribution. *p* < 0.05 was considered statistically significant.

For PK parameters, analysis of variance (ANOVA) was used to analyze the difference in the least-square means between denosumab and CMAB807 after the natural logarithmic transformation of C_max_, AUC_0-t_, and AUC_0-∞_. The GMRs and 90% CIs were obtained after conversion to an inverse natural logarithm. PK values were concluded to be bioequivalent between denosumab and CMAB807 if the 90% CIs for C_max_, AUC_0-t_, and AUC_0-∞_ were recorded to be between 80.00 and 125.00%. Notably, t_1/2_, CL/F and Vz/F were not included if AUC__%Extrap_ >20%. In addition, the Wilcoxon-signed rank test was used to compare the T_max_ of denosumab and its biosimilar.

## Results

### Subjects

**FIGURE 1:**
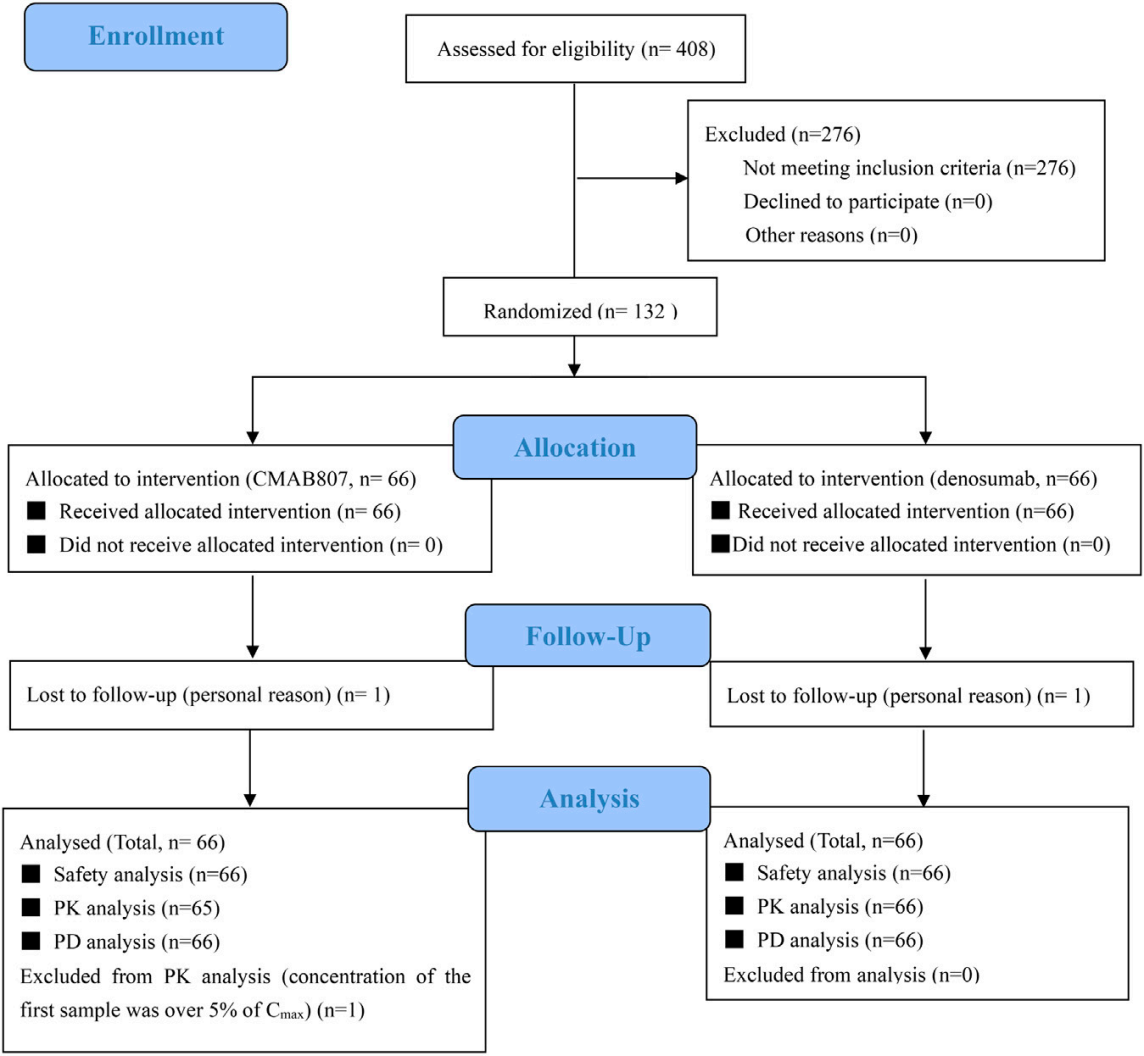
Flow chart of the study.

One hundred thirty-two subjects were enrolled in this study after screening 408 potential subjects. Sixty-six subjects were allocated to the CMAB807 group, and the other 66 subjects were allocated to the denosumab group. All 132 subjects were administered drugs, and at least one value for the PD dataset was collected; therefore, all subjects were included in the safety analysis and PD analysis sets. One subject in each group withdrew from the study for personal reasons. In addition, one subject (No. 1128) in the CMAB807 group was not included in the PK analysis set because his predose drug concentration was greater than 5% of C_max_. Therefore, 65 and 66 subjects were included in the PK analysis set in the CMAB807 and denosumab groups, respectively (Figure 1).

Demographic data are presented in Table 1. The mean age, height, body weight, and BMI were 27.6 years, 170.8 cm, 65.2 kg, and 22.3 kg/m^2^, respectively. The demographic parameters were not significantly different between the CMAB807 and denosumab groups (*p* > 0.05).

**TABLE 1:** Demographic characteristics of the study subjects.

Characteristic	CMAB807 group	Denosumab group	Total	*p* value
Subjects (n)	66	66	132	NA
Male, n (%)	66 (100%)	66 (100%)	132 (100%)	NA
Age (years)	27.3 ± 4.9	27.8 ± 4.9	27.6 ± 4.9	0.63
Height (cm)	171.1 ± 6.0	170.5 ± 5.0	170.8 ± 5.5	0.56
Weight (kg)	65.4 ± 7.6	65.0 ± 7.0	65.2 ± 7.3	0.85
BMI (kg/m^2^)	22.3 ± 1.9	22.3 ± 2.0	22.3 ± 1.9	0.94

Values are presented as the mean ± SD, unless specified otherwise.

BMI, body mass index.

### PK Evaluations

**FIGURE 2:**
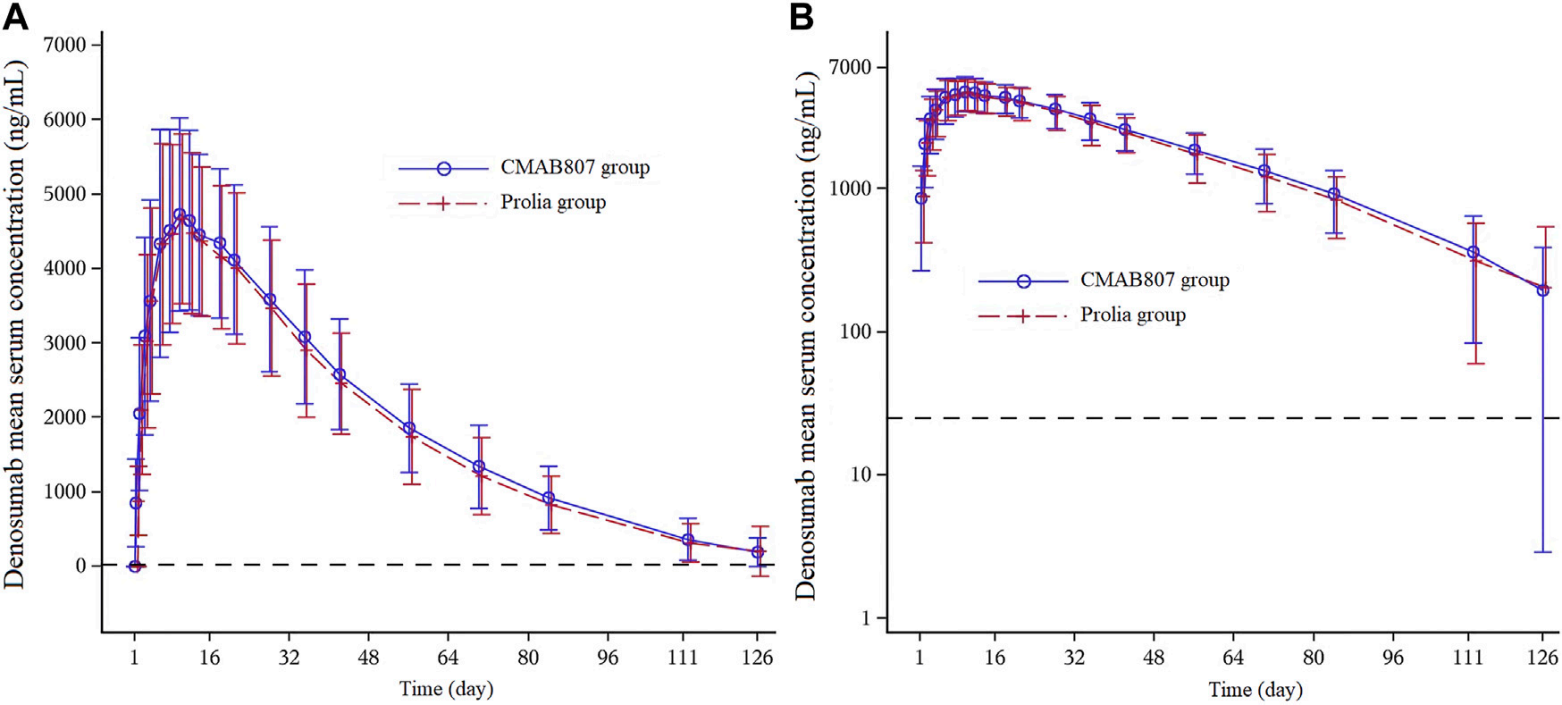
Mean serum concentration-time profiles following the SC injection of 60 mg of CMAB807 or denosumab in healthy Chinese male subjects **(A)**: Linear scale **(B)**: semilog scale. Note: the error bars indicate the standard deviations (SD); the dashed horizontal line refers to the LLOQ (25.0 ng/ml).

As shown in Figure 2, the entire PK profiles of CMAB807 and denosumab were similar. The PK parameters of denosumab and its biosimilar and the bioequivalence assessment of the PK parameters in this study are shown in Table 2. Both CMAB807 and denosumab were absorbed after a single-dose SC injection, with a median T_max_ of 9 days. Following T_max_, serum concentrations decreased biexponentially with an average apparent terminal t_1/2_ values of 17.2 and 18.0 days in the CMAB807 and denosumab groups, respectively.

**TABLE 2:** Pharmacokinetic parameters of CMAB807 and Prolia^®^ in each group.

Parameter	CMAB807 group (N = 65)	Denosumab group (N = 66)	*p* value	GMR (%, 90% CI)
C_max_ (μg/ml)	5.17 ± 1.33 (25.6)	5.03 ± 1.26 (25.0)	0.50	102.41 (94.68, 110.78)
AUC_0-t_ (h*μg/mL)	5,820 ± 1650 (28.4)	5,540 ± 1430 (25.9)	0.29	104.15 (95.95, 113.06)
AUC_0-∞_ (h*μg/mL)	6,000 ± 1780 (29.7)	5,710 ± 1540 (27.0)	0.99	103.89 (95.40, 113.12)
t_1/2_ (d)	17.2 ± 7.8 (45.1)	18.0 ± 12.5 (69.4)	0.26	
T_max_ (d)	9 [2, 20]	9 [2, 41]	0.77	
CL/F (ml/h)	11.0 ± 3.81 (34.6)	11.3 ± 3.09 (27.4)	0.85	
Vz/F (ml)	6,040 ± 2,320 (38.3)	6,720 ± 5,520 (82.2)	0.23	

Values are presented as the mean ± SD (CV%); T_max_ is reported as the median [min, max].; **Abbreviations:** GMR, geometric least-squares mean ratio; CI, confidence interval.

C_max_, AUC_0-t_ and AUC_0-∞_ are the primary bioequivalence evaluation parameters in this study. As shown in Table 2, following a single SC administration of CMAB807 or denosumab, the 90% CIs for the GMRs of C_max_, AUC_0-t_ and AUC_0-∞_ were fully contained within 80.00–125.00%, confirming the bioequivalence between them. In addition, all the primary and secondary PK parameters were comparable in the two groups (*p* > 0.05).

### PD Evaluations

**FIGURE 3:**
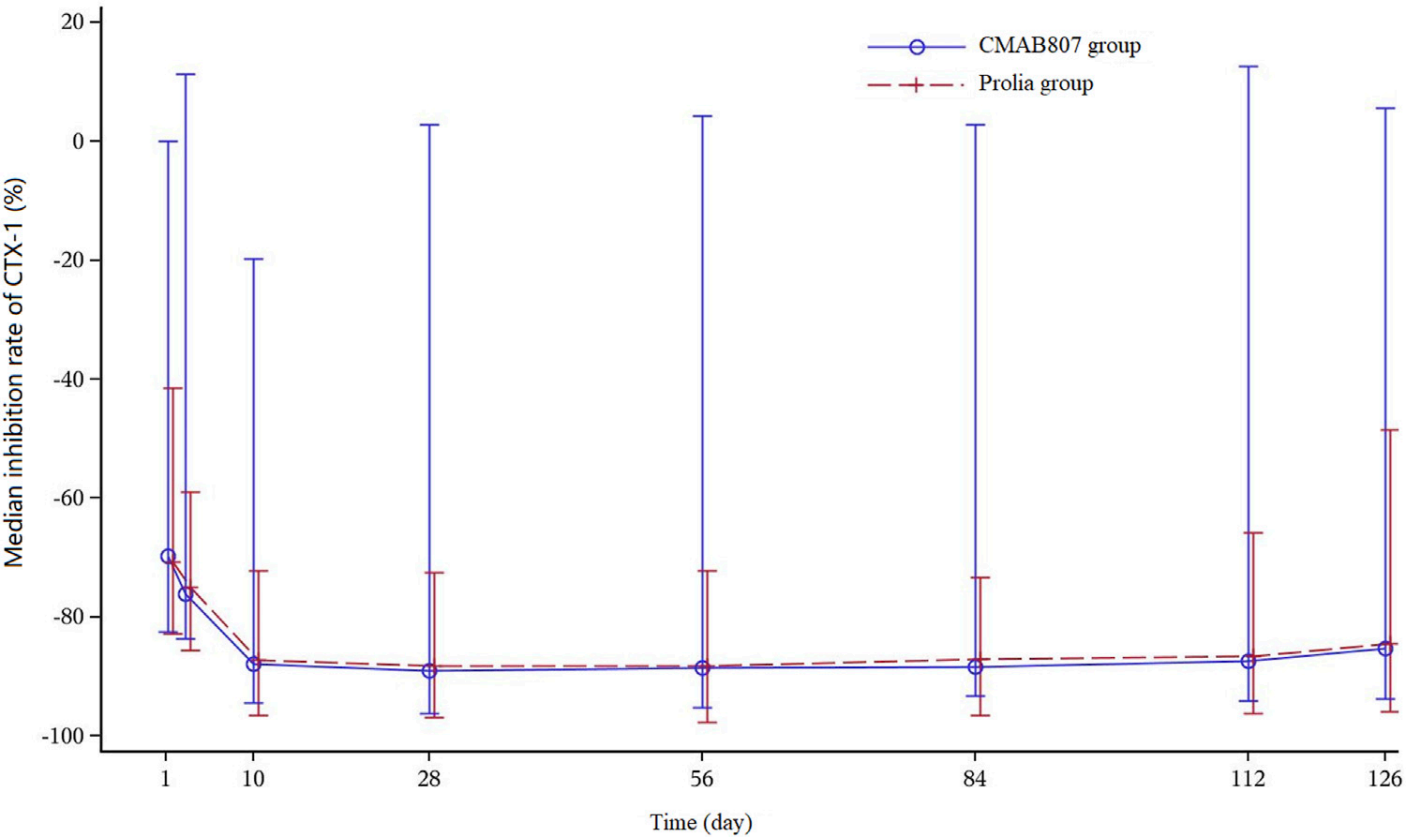
Median percent change in the CTX1-time profiles from baseline. Note: the error bars indicate the SD.

The median percent change in CTX levels from baseline versus time profiles after the administration of CMAB807 and denosumab are shown in Figure 3. The PD parameters (including I_min_, T_min_, I_max_, AUEC_0-t_, and AUEC_0–126 d_) and the PD profiles of the CMAB807 group and the denosumab group were similar (Table 3; Figure 3). CTX1 production was inhibited by up to approximately 85% (I_max_) after the administration of a single dose of denosumab or CMAB807 for 10 days. The median value of I_max_ was 648 h in both groups. The inhibition status was sustained for up to 126 days. The CTX1 reduction rate seemed to be attenuated at the end of the study. On day 126, the median CTX1 inhibition rates of the CMAB807 and denosumab groups were 82.8 and 82.4%, respectively.

**TABLE 3:** Pharmacodynamic parameters of CTX1 in each group (means ± SD (CV%) or medians [min, max]).

Parameter	CMAB807 group (n = 66)	Denosumab group (n = 66)	*p* value
I_min_ (ng/ml)	0.0839 ± 0.0261 (31.2)	0.0795 ± 0.0317 (39.9)	0.39
T_min_ (h)	648 [216, 2,660]	648 [216, 3,000]	0.92
I_max_ (%)	88.199 ± 9.3046 (10.5)	88.180 ± 4.6685 (5.3)	0.99
AUEC_0-t_ (hour*%)	259,000 ± 16,300 (6.3)	254,000 ± 21,400 (8.4)	0.21
AUEC_0-126d_ (hour*%)	260,000 ± 12,300 (4.7)	256,000 ± 15,400 (6.0)	0.13

Values are reported as the mean ± SD (CV%); T_max_ is reported as the median [min, max].

The PK and PD profiles indicate that the concentration-effect relationship for the two treatments is not different and that equivalent exposure results in an equivalent response, thus supporting their biosimilarity.

### Immunogenicity Evaluations

All subjects in the CMAB807 group were negative for ADA before dosing and throughout the study period. In the denosumab group, three subjects (No. 1004, 1011, and 1123) were positive for ADA before administration, and two subjects (No. 1011 and 1033) were ADA-positive after administration (on day 28 and day 84, respectively) but were negative at the end of the study. Therefore, the ADA characteristics between CMAB807 and denosumab were similar in this study.

### Safety Evaluations

Treatment-emergent adverse events (TEAEs) among 65 subjects (199 events, 98.5% [65/66]) in the CMAB807 group and 65 subjects (191 events, 98.5% [65/66]) in the denosumab group were recorded. No AEs led to death or discontinuation. No abnormal reactions at the injection site were observed. Most TEAEs were grade 1 or 2. Drug-related TEAEs (greater than grade 3 based on the CTCAE 5.0 criterion) were observed in three subjects in the CMAB807 group (increased ALT, AST, and triglyceride levels) and in one subject in the denosumab group (increased AST level). One serious adverse event (SAE) was reported in the CMAB807 group (open injury of the left hand because of an accident) and was estimated as grade 3, but not drug-related by the investigating physician. No drug-related SAE was reported (Table 4).

**TABLE 4:** TEAEs (safety set, n = 132).

Subjects with TEAEs	CMAB807 group		Prolia^®^ group		Total		*p* value
	n = 66		n = 66		n = 132		
	n (%)	[nAE]	n (%)	[nAE]	n (%)	[nAE]	
Any TEAE	65 (98.5%)	199	65 (98.5%)	191	130 (98.5%)	390	1.00
Drug-related TEAEs	64 (97.0%)	186	65 (98.5%)	173	129 (97.7%)	359	0.56
TEAEs over CTCAE grade 3	5 (7.6%)	7	1 (1.5%)	1	6 (4.5%)	8	0.14
Drug-related TEAEs over CTCAE grade 3	3 (4.5%)	3	1 (1.5%)	1	4 (3.0%)	4	0.39
SAEs	1 (1.5%)	0	0	0	1 (0.8%)	1	NA
Drug-related SAEs	0 (0%)	0	0 (0%)	0	0 (0%)	0	NA
Most common TEAEs by Preferred Term (≥5% of subjects in any of the treatment groups)							
*Laboratory examination*	63 (95.5%)	141	65 (98.5%)	124	128 (97.0%)	265	0.31
Increased parathyroid hormone level	61 (92.4%)	68	63 (95.5%)	69	124 (93.9%)	137	0.47
Increased alanine aminotransferase level	11 (16.7%)	12	6 (9.1%)	6	17 (12.9%)	18	0.19
Increased bilirubin level	10 (15.2%)	16	9 (13.6%)	11	19 (14.4%)	27	0.80
Increased aspartate amino transferase level	8 (12.1%)	10	2 (3.0%)	2	10 (7.6%)	12	0.05
Increased thyroid stimulating hormone level	8 (12.1%)	10	6 (9.1%)	6	14 (10.6%)	16	0.57
Increased free thyroxine level	4 (6.1%)	6	4 (6.1%)	5	8 (6.1%)	11	1.00
Increased blood leukocyte count	3 (4.5%)	3	4 (6.1%)	4	7 (5.3%)	7	0.70
Increased activated partial thromboplastin time	1 (1.5%)	1	4 (6.1%)	4	5 (3.8%)	5	0.17
Leukocytes detected in urine	1 (1.5%)	1	4 (6.1%)	4	5 (3.8%)	5	0.17
*Metabolic and nutritional diseases*	33 (50.0%)	45	26 (39.4%)	42	59 (44.7%)	87	0.22
Hypophosphatemia	16 (24.2%)	18	11 (16.7%)	13	27 (20.5%)	31	0.06
Hyperuricemia	8 (12.1%)	9	9 (13.6%)	12	17 (12.9%)	21	0.80
Hyperkalemia	7 (10.6%)	10	4 (6.1%)	6	11 (8.3%)	16	0.35
Hypocalcemia	4 (6.1%)	5	4 (6.1%)	5	8 (6.1%)	10	1.00
*Heart organ disease*	3 (4.5%)	3	8 (12.1%)	16	11 (8.3%)	19	0.12
I ventricular atrioventricular block	2 (3.0%)	2	5 (7.6%)	10	7 (5.3%)	12	0.24
Sinus bradycardia	1 (1.5%)	1	4 (6.1%)	5	5 (3.8%)	6	0.10
*Musculoskeletal and connective tissue diseases*	1 (1.5%)	1	4 (6.1%)	4	5 (3.8%)	5	0.17
Arthralgia	1 (1.5%)	1	4 (6.1%)	4	5 (3.8%)	5	0.17

Percentages are based on n.; **Abbreviations:** TEAE, treatment emergent adverse event; SAE, serious adverse event; n, number of subjects [nAE], the number times of the AE, occurred.

**FIGURE 4:**
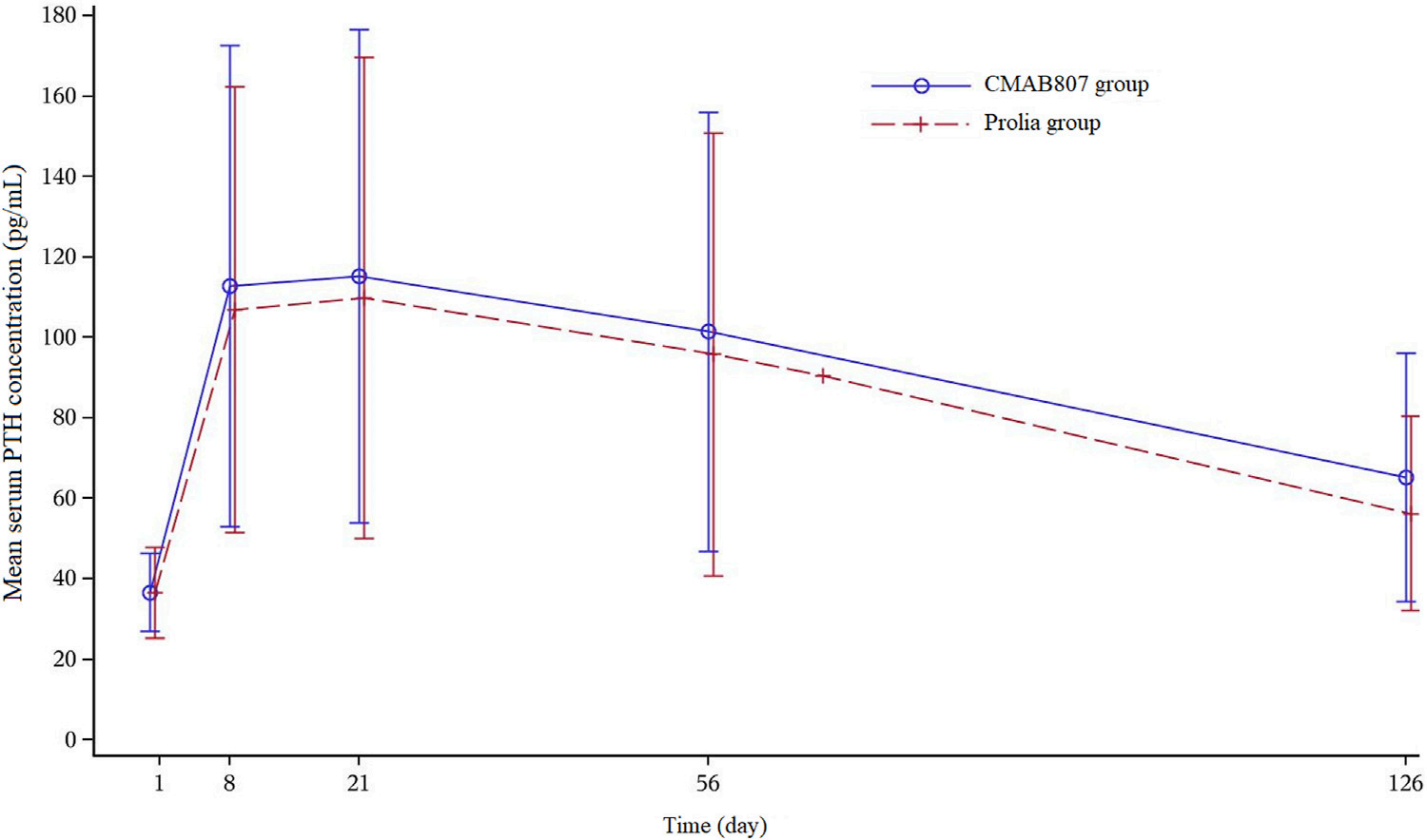
Mean serum PTH concentration versus time profiles after the SC injection of 60 mg of CMAB807 or denosumab in healthy Chinese male subjects. Note: the error bars indicate the SD.

All of the common TEAEs (≥5% of subjects in any of the treatment groups) were comparable in the two groups (*p* ≥ 0.05). The most common AEs in this study were increased parathyroid hormone (PTH) levels (92.4 vs 95.5%, CMAB807 vs Denosumab) (Table 4). Increased PTH levels were observed 8 days after administration and generally decreased after the 21st day. Most of the subjects returned to the baseline level at the end of the study without any treatment (Figure 4). The PTH profile was generally consistent with the PK characteristics of denosumab (median T_max_ = 9 days, range from 3 to 21 days). Among the subjects with elevated serum PTH levels, some also had hypophosphatemia, but very few had hypocalcemia. No clinically significant changes corresponding to the increased PTH levels were found during thyroid function detection. Thus, after the administration of 60 mg of CMAB807 or denosumab, the incidence of AEs that appeared in the two groups was similar, and both of the study drugs were considered generally safe and well tolerated.

## Discussion

Denosumab and its biosimilar (CMAB807) were generally well tolerated. The common TEAEs that occurred in this study were generally consistent with those listed on the label of denosumab, such as hypocalcemia, hypophosphatemia, increased PTH levels, and arthralgia (Amgen, 2021). The only SAE in the study was an “open injury of the hand” by accident in the CMAB807 group, which was judged as a nondrug-related AE. Furthermore, the number of drug-related TEAEs over CTCAE grade 3 in the CMAB807 group (3 events, which were increased ALT, increased AST and hypertriglyceridemia, respectively) was greater than the number in the Prolia^®^ group (1 event, which was increased AST). However, there were also subjects with increased ALT and hypertriglyceridemia in the Prolia^®^ group, just not to the extent of grade 3. The difference between the two groups was not statistically significant. The most common TEAE in this study was an increased PTH level (92.4% in the CMAB807 group and 95.5% in the denosumab group). The PTH level was temporarily increased by denosumab treatment. PTH is a critical regulator of skeletal development that promotes both bone formation and bone resorption (Li et al., 2020) and regulates calcium and phosphorus homeostasis (Civitelli and Ziambaras, 2011). The calcium status is strictly regulated by intestinal calcium absorption, bone resorption, and renal reabsorption (Nakamura et al., 2015). Therefore, denosumab strongly inhibits bone resorption, resulting in an increase in calcium release from the bones, causing hypocalcemia, followed by effects on the parathyroid gland, which is the reason for the increased serum PTH level and decreased serum phosphate level.

As the distribution and elimination of mABs are often proportional to body weight (Wang et al., 2008; Grant, 2009; Glassman and Balthasar, 2016), subjects were sorted according to their weights to reduce PK errors. However, a population PK meta-analysis of 22,944 serum-free denosumab concentrations in 495 healthy subjects and 1069 patients showed that the denosumab PK parameters do not depend on body weight (Sutjandra et al., 2011). Additionally, differences in exposure do not affect the response to denosumab (Chen et al., 2018). Therefore, a fixed dose (60 mg) for all patients is supported.

Overall, six subjects in the CMAB807 group and two subjects in the denosumab group received treatment for various adverse reactions. As denosumab and its biosimilar are recombinant mABs whose metabolic pathway usually involves the phagocytic function of mesenchymal cells rather than cytochrome P450 metabolism (Zhang et al., 2021), concomitant medications were considered to have little effect on the PK/PD characteristics of denosumab and its biosimilar.

Serum denosumab concentrations decreased at a faster rate when the serum denosumab concentration was less than approximately 1 μg/ml. The mechanism underlying this change in elimination rate is likely related to denosumab binding to RANKL (i.e., target-mediated disposition). This mechanism predominates at low serum denosumab concentrations (i.e., <1 μg/ml in this case) and becomes saturated as the serum denosumab concentration increases (An, 2020). A bioequivalence study confirmed that CMAB807 showed PK profiles similar to those of denosumab evaluated in healthy Chinese male subjects. The 90% CIs of the test-to-reference ratios for the primary PK endpoints, namely, C_max_, AUC_0-∞_ and AUC_0-last_, were observed to be within the predefined bioequivalence approved range of 80.00–125.00%, according to Chinese biosimilar guidelines (NMPA, 2015). The PK profiles in this study were also generally comparable to the profiles observed in Caucasian and Chinese populations in previous reports (Chen et al., 2018; Amgen, 2020; Zhang et al., 2021).

Denosumab administration resulted in significant inhibition of bone resorption, as assessed by CTX1 levels, which is a specific biomarker of bone reabsorption, whose serum concentration is suggestive of osteoclast activity (Williams and Sapra, 2021). In previous clinical studies of Caucasians, treatment with 60 mg of denosumab reduced CTX1 levels by approximately 70% in 6 h and 85% by 3 days (10 days in this study), with maximal reductions occurring by 1 month (Amgen, 2021). In the present study, the CTX1 level was reduced by 85% by 10 days, with a T_min_ of 27 days, which is comparable to previous results obtained from the Chinese population (Chen et al., 2018; Zhang et al., 2020). The administration of denosumab to Chinese patients appears to result in a less rapid reduction in CTX1 levels than in Caucasians. Reduced CTX1 levels were partially attenuated from a maximal reduction of ≥87% to ≥45% (range: 45–80%) at 180 days after administration, when serum denosumab levels were diminished, reflecting the reversibility of the effects of denosumab on bone remodeling (Amgen, 2021). However, in the present study, the recovery of the CTX1 inhibition rate was not fully observed because of the limited follow-up duration (126 days). The PK and PD similarities of CMAB807 and denosumab support further study of this biosimilar in phase III studies.

The immunogenicity and safety were analyzed. The overall ADA-positive rates were as low as 0% (0/66) in the CMAB807 group and 4.5% (3/66) and 3.0% (2/66) in the denosumab group before and after administration, respectively, which were similar to the positive rates reported in previous studies in both healthy subjects and patients (Amgen, 2021). The detection of ADAs does not significantly affect the PK parameters of denosumab, CTX1 inhibition rate (Zhang et al., 2020; Zhang et al., 2021), and clinically significant adverse reactions (FDA, 2010).

## Conclusion

In this phase I clinical study, the PK, PD and ADA profiles of the denosumab biosimilar (CMAB807) were similar to those of denosumab. The safety data were also comparable between the two groups. These results support the efficacy of CMAB807 as a denosumab biosimilar.

## Data Availability

The raw data supporting the conclusions of this article will be made available by the authors, without undue reservation.
